# Rapid Identification of Neonatal Sepsis Pathogens Using MALDI-TOF MS: Bacteriological Profile and Antimicrobial Susceptibility Patterns in a North Indian Tertiary Care Hospital

**DOI:** 10.7759/cureus.83436

**Published:** 2025-05-03

**Authors:** Rohan Acharya, Geeta Gathwala, Madhu Sharma

**Affiliations:** 1 Pediatrics, All India Institute of Medical Sciences, Bhubaneswar, Bhubaneswar, IND; 2 Neonatology, Pandit Bhagwat Dayal Sharma Post Graduate Institute of Medical Sciences, Rohtak, IND; 3 Microbiology, Pandit Bhagwat Dayal Sharma Post Graduate Institute of Medical Sciences, Rohtak, IND

**Keywords:** antimicrobial resistance, bacteriological profile, maldi-tof ms, neonatal sepsis, north india, rapid diagnostics

## Abstract

Background

Neonatal sepsis is a major cause of mortality in low- and middle-income countries (LMICs) such as India, where delayed diagnosis and antimicrobial resistance (AMR) challenge effective management. Conventional blood cultures, taking 2-5 days for pathogen identification, delay targeted therapy, worsening outcomes, and fueling AMR. Matrix-assisted laser desorption/ionization time-of-flight mass spectrometry (MALDI-TOF MS) (bioMérieux, USA) offers rapid diagnostics, potentially transforming neonatal sepsis care. This study assesses the diagnostic speed of MALDI-TOF MS versus conventional methods in neonates with suspected sepsis at a North Indian tertiary hospital, alongside bacteriological and AMR profiling.

Methodology

From November 2022 to November 2023, we prospectively enrolled 100 consecutive outborn neonates (<28 days) with suspected sepsis (positive sepsis screen: ≥2 parameters, e.g., C-reactive protein, leukocyte count) at a tertiary care hospital. Neonates with prior antibiotics, severe asphyxia, or malformations were excluded. Blood samples underwent BacT/Alert culture, with pathogens identified by conventional methods (2-5 days) and MALDI-TOF MS (VITEK MS, bioMérieux, USA). Time-to-identification was compared (paired t-test, p < 0.05). Antimicrobial susceptibility testing used VITEK-2 (bioMérieux, USA) per Clinical and Laboratory Standards Institute guidelines, with chi-square tests analyzing microbiological and AMR data.

Results

Among 100 neonates (50 early-onset sepsis (EOS), mean age = 1.34 ± 0.692 days; 50 late-onset sepsis (LOS), mean age = 14.418 ± 9.395 days), MALDI-TOF MS identified pathogens in 1.47 ± 0.979 days (median = 2 days) versus 2.83 ± 0.817 days (median = 3 days) for conventional culture (p = 0.0052), a 48% reduction. *Klebsiella pneumoniae* (35%) and *Acinetobacter baumannii* (28%) predominated EOS, while LOS included *K. pneumoniae* (30%), *A. baumannii* (30%), and *Pseudomonas aeruginosa* (10%, LOS-only). Gram-negative isolates showed >85% susceptibility to meropenem and ertapenem; amikacin and colistin were effective, though *P. aeruginosa* had reduced amikacin sensitivity (60%). Beta-lactams were poorly effective (<40%). Gram-positive isolates were sensitive to linezolid and vancomycin (>90%), except *Enterococcus* spp. (0% vancomycin susceptibility).

Conclusions

MALDI-TOF MS accelerates neonatal sepsis diagnosis by 1.36 days, enabling earlier targeted therapy. Coupled with dominant pathogens (*K. pneumoniae*, *A. baumannii*) and rising AMR (e.g., vancomycin-resistant *Enterococcus*), it highlights the need for rapid diagnostics in LMICs. Carbapenems remain viable empirical options, but resistance trends demand stewardship. This study supports integrating MALDI-TOF MS into neonatal care, enhancing outcomes and informing antibiotic policies.

## Introduction

Neonatal sepsis, defined as a bloodstream infection within the first 28 days of life, remains a critical global health issue, particularly in low- and middle-income countries (LMICs) such as India. The World Health Organization estimates that neonatal infections account for 1.6 million deaths annually, with 40% occurring in developing nations [1]. Survivors often face long-term neurodevelopmental impairments, amplifying morbidity [2]. Early diagnosis and timely initiation of effective antibiotic therapy are pivotal for improving outcomes. However, conventional blood culture methods, which typically require 2-5 days for pathogen identification, delay targeted treatment, increasing mortality risk and contributing to inappropriate antibiotic use, a key driver of antimicrobial resistance (AMR) [3]. The emergence of AMR among neonatal pathogens further complicates management, necessitating rapid, accurate diagnostics and region-specific surveillance.

In India, neonatal sepsis incidence varies widely, with blood culture-proven cases reported at 8.5 per 1,000 live births in 2002-2003 [4]. Risk factors include prematurity, low birth weight, maternal infections, and nosocomial transmission in resource-limited neonatal intensive care units (NICUs) [5]. Early-onset sepsis (EOS, <72 hours) often stems from vertical transmission, with pathogens like Group B *Streptococcus* (GBS) and *Escherichia coli *predominating, while late-onset sepsis (LOS, >72 hours) is typically nosocomial, involving organisms such as coagulase-negative staphylococci (CoNS) and *Klebsiella pneumoniae* [6,7]. Rapid pathogen identification can bridge the diagnostic gap, enabling prompt, tailored therapy and reducing AMR escalation.

This study evaluates the time-to-identification of pathogens using matrix-assisted laser desorption/ionization time-of-flight mass spectrometry (MALDI-TOF MS) (bioMérieux, USA) compared to conventional blood culture methods in neonates with suspected sepsis at a tertiary care hospital in North India, aiming to validate its rapid diagnostic potential in a resource-limited setting. By quantifying the time-to-identification and correlating it with bacteriological profiles and AMR patterns, we aim to demonstrate the clinical utility of rapid diagnostics in optimizing neonatal sepsis management. This approach addresses a critical gap in the current literature, offering actionable insights beyond reiterating known epidemiology.

## Materials and methods

This prospective, cross-sectional study was conducted from November 2022 to November 2023 at a tertiary care hospital in North India, involving the Departments of Pediatrics and Microbiology. Ethical approval was obtained from the Institutional Ethics Committee (approval number: IEC/2022/089), and informed parental consent was secured for all participants. We enrolled 100 consecutive outborn neonates (<28 days) presenting to the pediatric emergency with clinical suspicion of sepsis, based on a power calculation to detect a significant difference in diagnostic time (alpha = 0.05, power = 80%), considering the expected sepsis prevalence and resource limitations of a single-center study. Neonatal sepsis is defined by positive sepsis screens (≥2 parameters: C-reactive protein (CRP) = >10 mg/L, abnormal total leukocyte count = <5,000 or >15,000/mm³, absolute neutrophil count = <1,500/mm³, immature-to-total neutrophil ratio = >0.2, micro-erythryocyte sedimentation rate = >15 mm/hour) and requiring empirical antibiotics. Exclusion criteria included prior antibiotic use within 72 hours, severe birth asphyxia (APGAR <3 at five minutes or cord pH <7.0), or major congenital malformations, minimizing confounding factors and selection bias.

Blood samples (1-2 mL) were collected aseptically before antibiotic initiation and inoculated into BacT/Alert pediatric bottles (Becton Dickinson, USA). Positive cultures were subcultured onto blood agar and MacConkey agar, incubated at 37°C for 18-24 hours. Conventional identification relied on colony morphology, Gram staining, and biochemical tests, with results recorded over 2-5 days. Parallel aliquots were analyzed using MALDI-TOF MS (VITEK MS, bioMérieux, USA) per manufacturer protocols, targeting rapid identification within 24-48 hours. For CoNS, true pathogenicity was confirmed by ≥2 positive cultures plus clinical/laboratory evidence of sepsis (e.g., elevated CRP, lethargy, respiratory distress). Time-to-identification was calculated as the interval from sample inoculation to confirmed pathogen identification.

Antimicrobial susceptibility testing (AST) was performed using the VITEK-2 system (bioMérieux, USA), adhering to Clinical and Laboratory Standards Institute guidelines [8]. Susceptibility was expressed as the percentage of isolates sensitive to each antibiotic. Treatment decisions were guided by AST results and clinical status, with outcomes monitored until discharge or death.

Data were analyzed using SPSS version 25 (IBM Corp., Armonk, NY, USA). Descriptive statistics (mean ± SD, median, range) summarized time-to-identification and microbiological findings. Differences in culture duration between methods were assessed using the paired t-test, with p-values <0.05 indicating statistical significance. Chi-square tests evaluated associations between pathogen prevalence, sepsis type, and AMR patterns.

## Results

Of the 100 enrolled neonates, 50 had EOS (mean age = 1.34 ± 0.692 days; gestational age = 34.1 ± 0.935 weeks) and 50 had LOS (mean age = 14.418 ± 9.395 days; gestational age = 37.2 ± 1.235 weeks). Common symptoms included fever (32%), poor feeding (15%), and lethargy (11%) (Table 1).

**Table 1:** Demographic characteristics of neonates with sepsis. ^a^: mean ± standard deviation where applicable.

Variables	Values^a^
Early-onset sepsis	50
Late-onset sepsis	50
Age (days)	
Early sepsis	1.340 ± 0.692
Late sepsis	14.418 ± 9.395
Gestational age at birth (weeks)	
Early sepsis	34.1 ± 0.935
Late sepsis	37.2 ± 1.235
Birth weight (kg)	
Early sepsis	2.154 ± 0.788
Late sepsis	2.220 ± 0.680
Gender	
Female	57 (57%)
Male	43 (43%)
Delivery type	
Vaginal delivery	78 (78%)
Cesarean section	22 (22%)
Birth weight for gestational age	
Appropriate for gestational age	66 (66%)
Small for gestational age	34 (34%)
Rupture of membrane history (>24 hours)	33 (33%)
Fever in newborn	32 (32%)
Lethargy	11 (11%)
Poor feeding	15 (15%)
Seizure	17 (17%)

Diagnostic efficiency

Conventional blood culture identified pathogens in a mean of 2.83 ± 0.817 days (median = 3 days; range = 2-5 days), while MALDI-TOF MS achieved identification in a mean of 1.47 ± 0.979 days (median = 2 days; range = 1-5 days). The difference was statistically significant (p = 0.0052, paired t-test), indicating that MALDI-TOF MS reduced diagnostic time by approximately 48% (1.36 days). Notably, 78% of isolates were identified within two days using MALDI-TOF MS, compared to 32% with conventional methods (Table 2).

**Table 2:** Comparision between the turnaround time of MALDI-TOF and conventional blood culture. Statistical test: paired t-test; p-values <0.05 were considered significant. MALDI-TOF: matrix-assisted laser desorption/ionization time-of-flight

Variable	Conventional blood culture days (N = 100)	MALDI-TOF days (N = 100)	P-value
Mean	2.83	1.47	0.0052
Standard deviation	0.817	0.979	

Blood cultures from 100 neonates with sepsis, *Klebsiella pneumoniae* (33 isolates, 33%) and Acinetobacter *baumannii* (20 isolates, 20%) predominated, together accounting for 53% of cases. In early-onset sepsis (EOS, <72 hours, 50 neonates), *K. pneumoniae* was most frequent (14 isolates, 34.1% of 41 EOS positives), followed by *A. baumannii* (12 isolates, 29.2%), with *E. coli* (4 isolates, 9.8%) also notable. In late-onset sepsis (LOS, >72 hours, 50 neonates), *K. pneumoniae* led (19 isolates, 39.6% of 48 LOS positives), followed by *A. baumannii* (8 isolates, 16.7%) and *Pseudomonas aeruginosa* (7 isolates, 14.6%, exclusive to LOS). Methicillin-resistant *Staphylococcus aureus* (MRSA) was more common in LOS (6 isolates, 12.5%) than in EOS (2 isolates, 4.9%), while *Staphylococcus epidermidis* was less prevalent in both (2 isolates, 4.9% in EOS; 1 isolate, 2.1% in LOS). Detailed distributions are shown in Table 3. Of the 100 cultures, 41 were positive in EOS and 48 in LOS, with 9 and 2 culture-negative cases, respectively.

**Table 3:** Type and number of bacterial isolates in neonates with sepsis based on the onset of sepsis. Statistical test: paired t-test; p-values <0.05 were considered significant.

Microorganism	Number of isolates, n (%)		P-value
	Early onset, n (%)	Late onset, n (%)	
*Acinetobacter baumannii*	12 (29.2%)	8 (16.66%)	0.5071
*Klebsiella pneumoniae*	14 (34.14%)	19 (39.58%)	0.5028
*Staphylococcus hemolyticus*	3 (7.31%)	3 (6.25%)	1.0000
*Staphylococcus epidermidis*	2 (4.87%)	1 (2.08%)	0.6823
Methicillin-resistant *Staphylococcus aureus*	2 (4.87%)	6 (12.50%)	0.3099
*Pseudomonas stutzeri*	1 (2.43%)	0	0.4806
*Pseudomonas aeruginosa*	0	7 (14.58%)	0.0581
*Escherichia coli*	4 (9.75%)	2 (4.16%)	0.5597
*Burkholderia multivorans*	1 (2.43%)	0	0.4806
*Bacillus cereus*	2 (4.87%)	0	0.3173
*Streptococcus agalactiae*	0	1 (2.08%)	0.4806
*Enterococcus*	0	1 (2.08%)	0.4806
Total culture positives	41	48	
Total culture negatives	9	2	

Antimicrobial susceptibility

Among Gram-negative isolates, meropenem and ertapenem exhibited high susceptibility (>85%) for *K. pneumoniae*, *A. baumannii*, *E. coli*, and *P. aeruginosa*. Amikacin and colistin were effective against most Gram-negatives, though *P. aeruginosa* showed reduced sensitivity to amikacin (60%). Ciprofloxacin was highly effective against *A. baumannii* (90%) but less so against *K. pneumoniae* (50%). Beta-lactam antibiotics (cefepime, ceftazidime, ceftriaxone) showed poor efficacy (<40%) (Table 4). For Gram-positive isolates, linezolid and vancomycin were broadly effective (>90%), except against *Enterococcus* spp., where vancomycin susceptibility was 0%. Penicillins and cephalosporins were largely ineffective against staphylococci (<20%) (Table 5).

**Table 4:** Antibiotic sensitivity pattern of Gram-positive bacterial isolates. NT: not tested

Antibiotic	Sensitivity pattern, n (%)					
	*Staphylococcus* *aureus* (n = 8)	*Staphylococcus hemolyticus* (n = 6)	*Staphylococcus epidermidis* (n = 3)	*Streptococcus agalactiae* (n = 1)	*Enterococcus* (n = 1)	*Bacillus cereus* (n = 2)
Gentamicin	7 (87.5%)	5 (83.3%)	3 (100%)	1 (100%)	NT	0
Imipenem	4 (50%)	NT	NT	NT	NT	2 (100%)
Meropenem	4 (50%)	NT	NT	NT	NT	2 (100%)
Piperacillin/Tazobactam	5 (62.5%)	2 (33.4%)	NT	NT	NT	2 (100%)
Tigecycline	5 (62.5%)	5 (83.3%)	3 (100%)	1 (100%)	NT	2 (100%)
Ciproﬂoxacin	6 (75%)	4 (66.7%)	3 (100%)	0	0	2 (100%)
Amikacin	NT	NT	NT	NT	NT	0
Cefoperazone/Sulbactam	0	0	0	0	NT	2 (100%)
Ertapenem	4 (50%)	NT	NT	NT	NT	2 (100%)
Linezolid	7 (87.5%)	5 (83.3%)	3 (100%)	1 (100%)	1 (100%)	0
Fosfomycin	NT	NT	NT	NT	NT	NT
Cefepime	2 (25%)	1 (16.7%)	NT	NT	NT	2 (100%)
Cotrimoxazole	2 (25%)	2 (33.4%)	3 (100%)	1 (100%)	NT	NT
Teicoplanin	4 (50%)	2 (33.4%)	3 (100%)	1 (100%)	NT	0
Vancomycin	4 (50%)	6 (100%)	3 (100%)	1 (100%)	0	0
Tetracycline	3 (37.5%)	3 (50%)	3 (100%)	1 (100%)	0	0
Amoxyclav	0	0	0	0	0	0
Ceftazidime	0	1 (16.7%)	0	0	NT	0
Ceftriaxone	0	0	0	0	NT	2 (100%)
Doxycycline	2 (25%)	2 (33.4%)	3 (100%)	1 (100%)	0	NT
Levoﬂoxacin	2 (25%)	3 (50%)	3 (100%)	NT	NT	NT
Clindamycin	2 (25%)	4 (66.7%)	3 (100%)	1 (100%)	NT	0
Erythromycin	1 (12.5%)	4 (66.7%)	3 (100%)	0	0	0
Cefotaxime	0	0	0	0	NT	2 (100%)
Cefuroxime	0	0	0	0	NT	2 (100%)

**Table 5:** Antibiotic sensitivity pattern of the Gram negative bacterial isolates. NT: not tested

Antibiotic	Sensitivity pattern, n (%)				
	*Klebsiella pneumoniae* (n = 33)	*Acinetobacter* (n = 20)	*Escherichia coli* (n = 6)	*Pseudomonas* (n = 8)	*Burkholderia* (n = 1)
Gentamicin	26 (78.8%)	17 (85%)	6 (100%)	4 (50%)	1 (100%)
Imipenem	33 (100%)	18 (90%)	6 (100%)	5 (62.5%)	0
Meropenem	32 (96.9%)	20 (100%)	6 (100%)	5 (62.5%)	1 (100%)
Piperacillin/Tazobactam	26 (78.8%)	17 (85%)	5 (83.3%)	5 (62.5%)	1 (100%)
Tigecycline	26 (78.8%)	17 (85%)	4 (66.7%)	0	0
Ciproﬂoxacin	17 (51.5%)	19 (95%)	3 (50%)	4 (50%)	1 (100%)
Amikacin	27 (81.8%)	17 (85%)	5 (83.3%)	8 (100%)	1 (100%)
Cefoperazone/Sulbactam	26 (78.8%)	15 (75%)	5 (83.3%)	7 (87.5%)	0
Colistin	29 (87.9%)	14 (70%)	6 (100%)	4 (50%)	NT
Ertapenem	29 (87.9%)	11 (55%)	6 (100%)	5 (62.5%)	NT
Linezolid	9 (27.2%)	10 (50%)	4 (66.7%)	4 (50%)	0
Fosfomycin	22 (66.6%)	12 (60%)	4 (66.7%)	2 (25%)	0
Cefepime	15 (45.5%)	9 (45%)	5 (83.3%)	6 (75%)	0
Cotrimoxazole	15 (45.5%)	6 (30%)	4 (66.7%)	3 (37.5%)	0
Teicoplanin	7 (21.2%)	10 (50%)	2 (33.3%)	1 (12.5%)	0
Vancomycin	8 (24.2%)	10 (50%)	0	1 (12.5%)	0
Tetracycline	8 (24.2%)	9 (45%)	0	0	NT
Amoxyclav	18 (54.5%)	0	2 (33.3%)	0	0
Ceftazidime	1 (3.03%)	7 (35%)	1 (16.7%)	0	1 (100%)
Aztreonam	2 (6.06%)	7 (35%)	0	0	1 (100%)
Ceftriaxone	4 (12.1%)	2 (10%)	0	2 (25%)	0
Doxycycline	NT	1 (5%)	NT	NT	NT
Levoﬂoxacin	NT	3 (15%)	NT	NT	NT
Cefotaxime	3 (9.09%)	2 (10%)	0	0	0
Cefuroxime	3 (9.09%)	2 (10%)	0	0	0

## Discussion

This study underscores the transformative potential of MALDI-TOF MS (bioMérieux, USA) in neonatal sepsis management by significantly accelerating pathogen identification. The 1.36-day reduction in diagnostic time (p = 0.0052) compared to conventional methods aligns with prior reports [1,9] and offers a novel contribution beyond existing epidemiological data. Rapid identification enables earlier initiation of targeted therapy, potentially reducing mortality, morbidity, and AMR development, critical in LMICs where delayed diagnosis exacerbates outcomes [10]. Unlike previous studies focusing solely on pathogen prevalence [11], our integration of diagnostic speed with microbiological profiling addresses a practical gap in neonatal care.

The bacteriological findings revealed *K. pneumoniae* and *A. baumannii* as dominant pathogens, consistent with regional trends [12]. The prominence of *K. pneumoniae* in both EOS and LOS contrasts with historical GBS dominance in EOS [13], suggesting evolving epidemiology possibly linked to maternal colonization or nosocomial spread. LOS isolates, including *P. aeruginosa* and MRSA, reflect NICU-related risks, corroborating prior observations [14]. Discrepancies with earlier local data reporting *P. aeruginosa* predominance [15] may stem from improved infection control or shifting resistance patterns.

AMR patterns highlight a concerning trend, with *K. pneumoniae* exhibiting high resistance to third-generation cephalosporins (>60%), consistent with global reports [16]. Carbapenems (meropenem, ertapenem) and colistin remain effective, supporting their role in empirical therapy pending AST results [17]. However, the emergence of vancomycin-resistant *Enterococcus* signals an urgent need for stewardship. The rapid diagnostics afforded by MALDI-TOF MS can mitigate overuse of broad-spectrum agents, aligning treatment with susceptibility profiles sooner.

Limitations and generalizability

While our sample size (n = 100) and single-center design limit broad extrapolation, the consecutive enrollment and strict inclusion criteria reduce selection bias compared to retrospective studies. The cost-effectiveness of MALDI-TOF MS was not assessed, limiting its applicability to smaller hospitals. The focus on outborn neonates reflects real-world referral patterns in LMICs, enhancing relevance. Future multicenter studies should be conducted to validate these findings across diverse settings.

## Conclusions

By demonstrating the diagnostic speed of MALDI-TOF MS, this study advocates its integration into neonatal sepsis protocols, particularly in resource-constrained regions. Coupled with local AMR surveillance, it can guide empirical therapy, curb resistance, and inform policy. These insights extend beyond reiterating known data, offering an actionable advancement in clinical practice.
